# Perspective on Protein Arginine Deiminase Activity—Bicarbonate Is a pH-Independent Regulator of Citrullination

**DOI:** 10.3389/fimmu.2018.00034

**Published:** 2018-01-18

**Authors:** Yebin Zhou, Nanette Mittereder, Gary P. Sims

**Affiliations:** ^1^Department of Respiratory, Inflammation, and Autoimmunity, MedImmune LLC, Gaithersburg, MD, United States

**Keywords:** neutrophil, citrullination, bicarbonate, PAD2, PAD4

## Abstract

Protein citrullination catalyzed by peptidyl arginine deiminase (PADs) is involved in autoimmune disease pathogenesis, especially in rheumatoid arthritis. Calcium is a key regulator of PAD activity, but under normal physiological conditions it remains uncertain how intracellular calcium levels can be raised to sufficiently high levels to activate these enzymes. In pursuit of trying to identify other factors that influence PAD activity, we identified bicarbonate as a potential regulator of PAD activity. We demonstrate that physiological levels of bicarbonate upregulate citrullination by recombinant PAD2/4 and endogenous PADs in neutrophils. The impact of bicarbonate is independent of calcium and pH. Adding bicarbonate to commercial PAD activity kits could increase assay performance and biological relevance. These results suggest that citrullination activity is regulated by multiple factors including calcium and bicarbonate. We also provide commentary on the current understanding of PAD regulation and future perspective of research in this area.

## Introduction

Protein citrullination (or deimination) is the conversion of peptidylarginine to citrulline. This process is mediated by a family of enzymes; peptidyl arginine deiminase (PADs), including five isozymes in human (PAD1-4, PAD6) (1–3). These isozymes are expressed in different tissues and are involved in distinct functions. Among them, PAD2 and PAD4 are expressed mainly in leukocytes, especially enriched in neutrophils and are widely studied in immune dysregulations. Elevated protein citrullination was observed in inflamed joints of patients with rheumatoid arthritis (RA) (4–7). Autoantibodies against citrullinated proteins (ACPA) are present in approximately 70% of patients with RA and are highly diagnostic for the disease (8–10).

Of the five arginine deiminases, only PAD4 contains a nuclear localization sequence and its main physiological function appears to be histone citrullination (11, 12). Citrullination regulates binding of histone 1 to chromatin, alters the gene expression, and regulates stem cell differentiation (13). According to some reports, histone hypercitrullination leads to chromatin decondensation and the formation of neutrophil extracellular trap (NET formation) (12, 14). PAD4 activity also regulates antimicrobial defense in soft tissue infections (14). Increased NET formation is evident and considered pathogenic in systemic lupus erythematosus and ANCA-associated vasculitis (15–20). PAD4 and citrullinated proteins are found in NETs, so, NET formation may be a critical source of citrullinated autoantigen in RA (21–24). Neutrophil hypercitrullination mediated by calcium influx was also found in synovial fluid from patients with RA (25). Consequently, PADs are being increasingly recognized as therapeutic targets (26). Inhibition of PAD activity suppressed pathology in murine models of autoimmune disease (27–30). These effects have been attributed mainly to the inhibition of PAD4 (31).

Recent studies have focused on PAD activity in pathological conditions; however, little is known about the physiological functions of PADs, and how these enzymes are regulated at a biochemical level (32). It is clear that calcium ions are essential for PAD activity. X-ray crystallography has revealed that PAD4 binds to five calcium ions (33). Two calcium ions are in the C-terminal domain of PAD4 at the bottom of the active domain. The binding of those two calcium ions to the acidic concave surface of the C-terminal domain is crucial for recognition of the substrate and PAD activity (33). Another three calcium ions reside on the molecular surface of the PAD4 N-terminal domain, and these ions may stabilize protein structure and are important for the full activation of PAD4 (34). Other than calcium, reduced glutathione (GSH) was recently described as an *in vivo* PAD co-activator to maintain the reduced state of Cys645 in PAD4 catalytic domain (Cys647 in PAD2) (35). There could well be an oxidation/reduction (redox) balance regulation of PAD activity (36).

Here, we demonstrated that bicarbonate is important for optimal recombinant PAD2/4 activity. Neutrophil histone citrullination and hypercitrullination at physiological calcium concentrations was also regulated by bicarbonate. The impact of bicarbonate on citrullination was observed at different calcium concentrations and was independent of pH. Our work has revealed a previously unknown role of bicarbonate on PAD activity regulation. Based on these findings, we propose that *in vivo* PAD activity could be under complex regulation by factors including calcium, bicarbonate, and redox.

## Recombinant PAD2/4 Require Bicarbonate for Optimal Histone Citrullination

**Figure 1:**
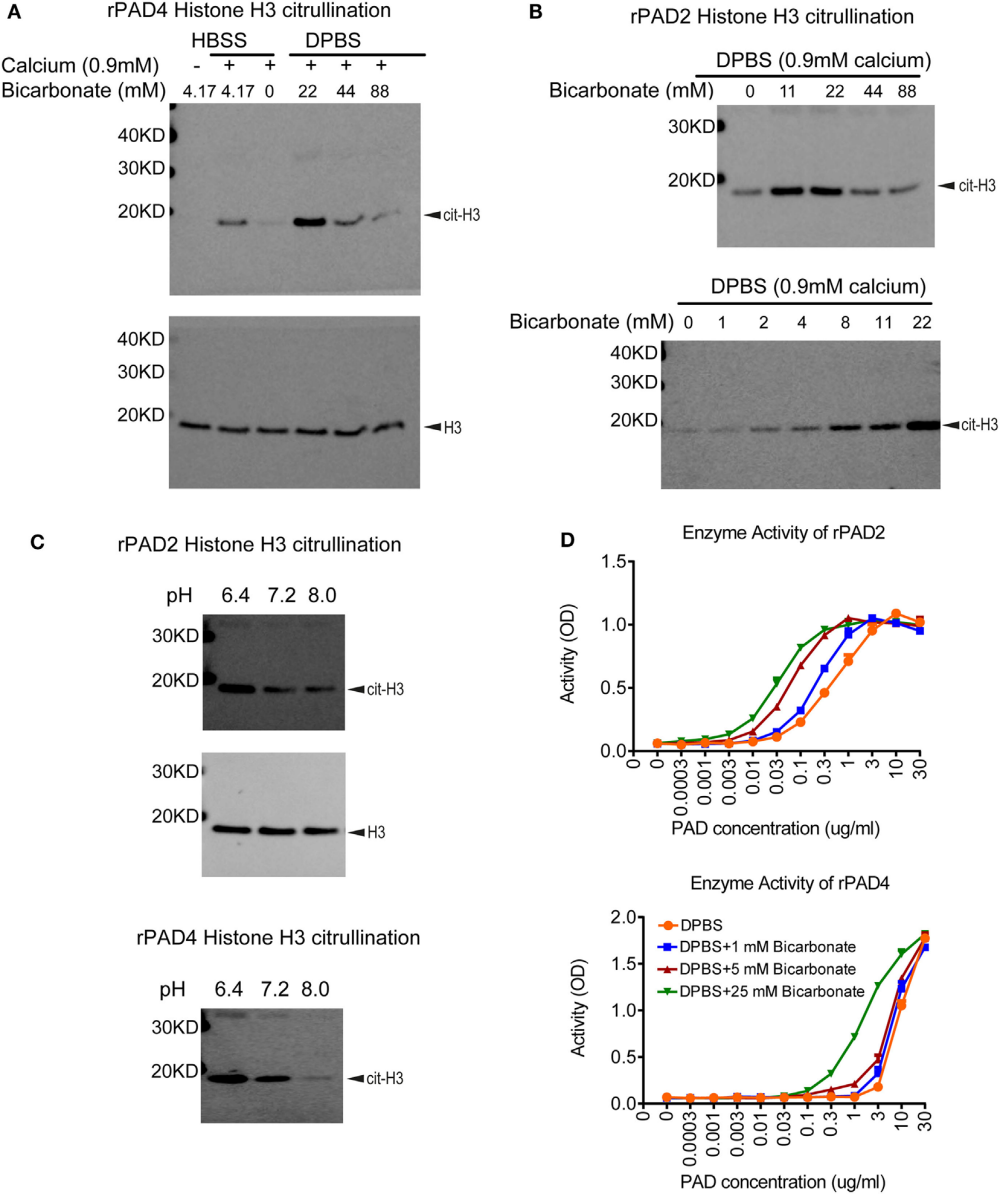
Bicarbonate impacts recombinant protein arginine deiminase (PAD) activity. **(A)** Histone H3 citrullination by recombinant human PAD4 for 1 h at 37°C with 5% CO_2_ in HBSS or DPBS with different bicarbonate concentrations. Total histone H3 was used as loading control. **(B)** Histone H3 citrullination by recombinant human PAD2 for 1 h at 37°C with 5% CO_2_ in DPBS with different bicarbonate concentrations. Results are representative of three independent experiments. **(C)** Histone H3 citrullination by recombinant human PAD2/4 for 1 h at 37°C with 5% CO_2_ in DPBS with 0.9 mM calcium under different pH levels in the absence of bicarbonate. Total histone H3 was used as loading control. **(D)** Recombinant human PAD2/4 activity in DPBS with different bicarbonate concentrations measured by ELISA-based fibrinogen citrullination assay. *r*^2^ values of nonlinear regression curve fit were higher than 0.98 for all conditions. Significant increase of PAD activity in DPBS with 25 mM bicarbonate was confirmed with two-way ANOVA analysis (*P* < 0.0001).

To assess inhibitors of PAD activity, various assays are in use, which consist of recombinant enzyme, a simple buffer or tissue culture media (e.g., Tris–HCl pH 7–8, HBSS, RPMI, or DMEM), calcium, a reduction reagent, and an arginine containing substrate. A biochemical or antibody-based approach is then used to detect the citrullinated substrate. One intriguing difference between these citrullination assays is the amount of bicarbonate included in the buffer. Human serum contains 17–29 mM bicarbonate (37), whereas Tris–HCl buffer contains no bicarbonate, HBSS, RPMI, and DMEM media contain 4.17, 23.81, and 44.05 mM of bicarbonate, respectively. Initially, we verified the importance of calcium for PAD activity in a histone H3 citrullination assay containing HBSS (Figure 1A). We next assessed the impact of bicarbonate on PAD4 and PAD2 activity on histone H3 citrullination in DPBS/HEPES (adjusted to pH 7.2) with 0.9 mM calcium. Increased citrullination was detected with both recombinant PAD4 and PAD2 in the presence of low amounts of bicarbonate (Figures 1A,B). The requirement for bicarbonate appeared greater for PAD4 than PAD2 activity, since minimal histone H3 citrullination was observed with PAD4 in the absence of bicarbonate (Figure 1A). PAD2 activity increased with increasing amounts of bicarbonate from 1 to 22 mM (Figure 1B). Since the pH was maintained at a consistent pH 7.2 in these assays, the results could indicate that the impact of bicarbonate on recombinant PAD activity was independent of pH.

To further confirm the pH-independent effect of bicarbonate and explore the effect of pH on PAD activity, we performed histone H3 citrullination with recombinant enzyme under different pH levels adjusted by HCl and NaOH. Both PAD2 and PAD4 showed higher activity at pH of 6.4 compared to 7.2 and 8.0 (Figure 1C). Our data suggest that, for PAD2/4, their optimal pH for histone citrullination could be at a more acidic condition. Since higher bicarbonate levels would increase pH (without additional adjustment), these results confirm that bicarbonate increases PAD activity independent of pH.

## Bicarbonate Improves PAD Activity in ELISA-Based Fibrinogen Citrullination Assays

To verify that the impact of bicarbonate was not limited to histone H3 citrullination or related to the western blot detection method, we examined the impact of bicarbonate on an ELISA-based fibrinogen citrullination assay, again using DPBS/HEPES (adjusted to pH7.2) with 0.9 mM calcium. Bicarbonate significantly increased fibrinogen citrullination by PAD2 and PAD4 in a dose-dependent manner (Figure 1D). These results demonstrate that the impact of bicarbonate on PAD activity was independent of substrates or detection methods and provide additional supportive evidence that bicarbonate regulates recombinant PAD activity.

## Bicarbonate Impacts Neutrophil Histone H3 Citrullination

**Figure 2:**
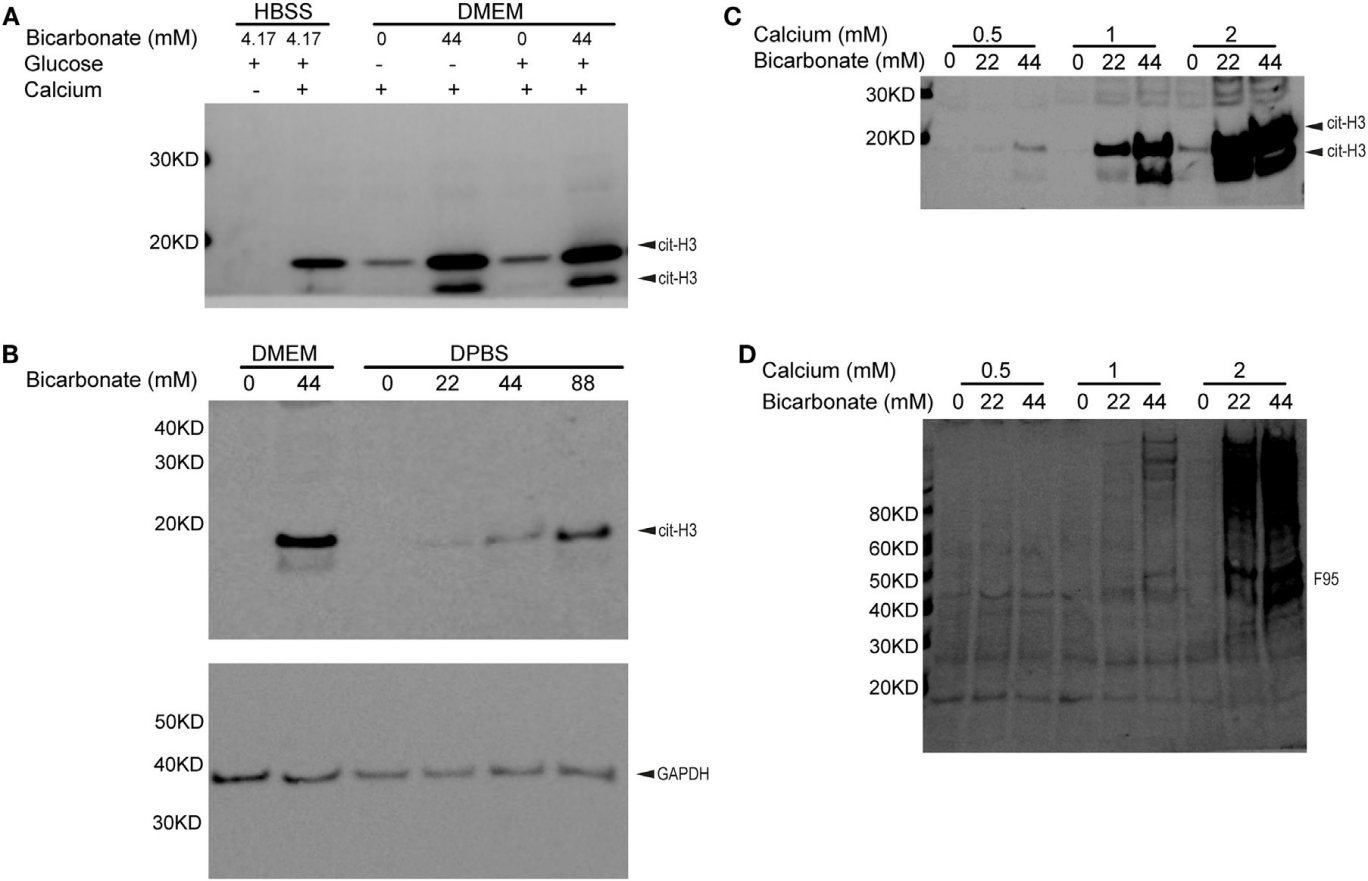
Bicarbonate level impacts neutrophil citrullination. **(A)** Ionomycin-induced neutrophil histone H3 citrullination with HBSS and DMEM (30 min at 37°C with 5% CO_2_). **(B)** Bicarbonate dose–response of neutrophil histone H3 citrullination stimulated by ionomycin (30 min at 37°C with 5% CO_2_). GAPDH used as loading control. **(C)** Ionomycin-induced neutrophil histone H3 citrullination with different bicarbonate and calcium levels. **(D)** Neutrophil hypercitrullination detected by anti-citrulline antibody F95 of panel **(C)**. Results are representative of three different donors.

To determine if bicarbonate impacts endogenous PAD activity, we examined neutrophil citrullination induced by ionomycin-mediated calcium influx. Interestingly, in the presence of calcium, ionomycin-treated neutrophils in DMEM showed much stronger histone H3 citrullination when bicarbonate was present (pH was adjusted to 7.2 with HEPES in all DMEM media, Figure 2A). A previous study suggested that high glucose in diabetes was associated with elevated neutrophil PAD4 expression and NET formation (38). However, we found that added extracellular glucose did not affect ionomycin/calcium-induced citrullination (Figure 2A). This suggests that glucose does not impact PAD activity directly.

We confirmed that neutrophil histone citrullination was also increased with bicarbonate in DPBS, a much simple buffer without vitamins, amino acids, and other factors present in tissue culture media (Figure 2B).

Taken together, we conclude that bicarbonate promotes intracellular PAD activity.

## Bicarbonate Impacts Neutrophil Citrullination at Different Calcium Levels

Since high levels of calcium are critical for PAD activity, we sought to determine if bicarbonate could promote citrullination independent of calcium concentrations. Bicarbonate increased neutrophil histone H3 citrullination (Figure 2C) and hyper citrullination (Figure 2D) at three different calcium concentrations from 0.5 to 2 mM. pH was adjusted to 7.2 with HEPES in DPBS and DMEM used. These results demonstrate that bicarbonate promotes intracellular citrullination at sub-physiological and physiological levels of serum calcium. Although there is a requirement for calcium for citrullination, bicarbonate independently increases PAD activity.

## Discussion

Current research on PAD biology has focused on the role of citrullination in disease pathogenesis and efforts to inhibit them. Little progress has been made on how PADs are activated and their functions under non-pathogenic conditions. The only essential factor known for PAD enzyme activity is calcium (1), although a reducing environment is also required to prevent oxidation of cysteine in the catalytic domain (36). We show for the first time that bicarbonate also has a profound impact on the activity of recombinant PAD enzymes and intracellular citrullination in neutrophils. The increased PAD activity promoted by bicarbonate is independent of its pH buffering capacity, and it acts independently of calcium and glucose. Therefore, bicarbonate may function as an important regulator of PAD activity.

Bicarbonate is the main buffering component in blood and an important *in vivo* signaling molecule (37, 39). Human serum contains 17–29 mM bicarbonate, which maintains serum pH levels at 7.4 (37). Bicarbonate and CO_2_ concentrations are also essential in maintaining optimal tissue culture growth conditions (40). Because of its important role in pH buffering, it is essential to separate the impact of pH and bicarbonate in biological functions. To focus on the impact of bicarbonate only in citrullination, we used HEPES to adjust all media pH to 7.2, then added different concentrations of bicarbonate. These results were consistent with different media such as DMEM, RPMI (data not shown), and DPBS that contained different inorganic ions and other weak acid and base-buffering pairs. The effect of bicarbonate on citrullination was consistent across different media and buffers, further demonstrated that the impact was indeed due to bicarbonate, independent of pH, and not from many other components in the tissue culture media.

Interestingly, we observed that recombinant histone H3 citrullination was increased by both recombinant PAD2 and PAD4 at a mildly acidic pH of 6.4. This further confirmed the impact of bicarbonate was independent of pH since higher bicarbonate leads to more alkaline pH levels. Previous studies with a different substrate—Benzoyl l-arginine ethyl ester (BAEE) showed optimal pH of 7.6 for PAD4 (41). This difference is likely due to the different substrates used in experiments, as BAEE, unlike histone, is a small molecule substrate.

Leppkes et al. first discovered that pancreatic juice could induce NET formation (42). Bicarbonate is a main component in pancreatic juice and was found to increase NET formation through histone citrullination. Later studies have shown that bicarbonate in media could impact NET formation through pH (43, 44). We limited the stimulation time in neutrophil citrullination assays to 30 min to focus on PAD enzymatic activity rather than downstream NET formation. The impact of bicarbonate on PAD2 and PAD4 activity was independent of pH and substrate. It remains possible that other pathways involved with NET formation may be impacted by bicarbonate through increased pH.

We showed that bicarbonate greatly impacted citrullination in biochemical assays with recombinant PADs and in neutrophils. The profound interest in PADs has led to an increase in different citrullination assays (45, 46). Those assays consider calcium levels and the reducing environment. All PADs require high calcium levels to be active (1), and the PAD catalytic domain contains a critical cysteine, which requires a reducing environment to maintain proper enzymatic function (35). Bicarbonate is a common but sometimes overlooked component in tissue culture media, which is necessary for optimal PAD activity. Our work suggests that a reconfiguration of assay buffers is needed to reflect the biology of citrullination.

More importantly, our study has indicated that the regulation of PAD activity is rather more complicated than previously known. Both bicarbonate and calcium can act as physiological signaling molecules. Remarkably, there are few examples of how bicarbonate regulates enzyme activity. A well-characterized example of this would be soluble adenylyl cyclase (sAC) (47). sAC is the only intracellular adenylyl cyclase that produces cAMP in response to calcium or bicarbonate signals (48, 49). sAC is conserved from cyanobacteria to mammals, with a wide range of functions including neuron activation to spermatozoa (50, 51). Intriguingly, the crystal structure showed that bicarbonate binds adjacent to Arg176 in human sAC, which acts as a switch that enables formation of the catalytic cation sites (52, 53). It is interesting to speculate that PADs could function by a similar process to sAC with citrullination regulated as a potential secondary messenger as seen with cAMP. Future crystallography studies are needed to understand whether bicarbonate impacts PAD structure, and how it could regulate citrullination activity.

In conclusion, we demonstrate that bicarbonate is a potential regulator of PAD activity. PADs could well be under a complex web of regulating factors including calcium and bicarbonate, responding to different *in vivo* signals through specific citrullination events. Dissecting those regulating signals, citrullination events and functional consequences could unlock a new level understanding of biology far beyond autoimmunity.

## Perspective on Future PAD Biology

Despite the focus on PADs and citrullination in the context of autoimmune diseases, there remains much to learn about the normal physiological functions of PADs. We recently reported that human neutrophils express active PAD4 on the surface and secrete active PAD2 (54). The role of extracellular PADs remains undefined but may modulate extracellular protein functions and fine tune inflammation. Within the cell, nuclear-located PAD4 could also regulate gene expression in concert with other histone modification enzymes (13, 55, 56). These physiological functions would require a precise regulation of PAD activity.

Although we showed that bicarbonate can impact citrullination independent of calcium and pH, there are still important gaps in our understanding of PAD regulation. For example, the millimolar concentrations of calcium required for PAD activation do not normally exist intracellularly even following cellular activation (25, 54, 57). So, how can PAD function inside a cell with nanomolar level intracellular calcium? It is plausible that high calcium concentration may exist in localized regions/specific organelles within a cell where PADs could be active. Alternatively, PADs could have other intracellular binding partners or co-factors that lower the calcium threshold for activation. Indeed, it has been shown that anti-PAD3/4 autoantibodies from patients with RA can increase PAD activity in low calcium conditions (58, 59). This calcium paradox for PAD activity is critical for our understanding of intracellular PAD activity, is largely overlooked, and requires further investigation.

Remarkably, intracellular protein citrullination is mostly observed with large calcium influx under membrane lytic conditions (25, 57, 60, 61). Detection of intracellular citrullination under normal healthy conditions is challenging. This may be a consequence of the limited citrullination detection methods and their low sensitivity. Improvements in technology may enable better assessment of intracellular citrullination. Alternatively, we should also consider the possibility that PADs may have functions other than citrullination. It is notable that besides the cysteine present at the catalytic domain (35, 36), PAD2 and PAD4 both exhibit in excess of 10 free surface exposed cysteines. This remarkably unusual characteristic must have some physiological relevance, whether this is related to binding substrate, or some other function such as acting as scavenger for ROS, remains to be determined. In any case, it is clear that there remains much to learn about the regulation and function of PADs in health and disease, and future research efforts will inevitably unravel new and exciting biology.

## Materials and Methods

### Human Donors and Neutrophil Isolation

Blood from healthy volunteers was obtained with informed consent under MedImmune, LLC’s blood donation program, and studies using human cells were performed in accordance with the Institutional Review Board guidelines. Neutrophils were isolated from heparin anticoagulated blood on a discontinuous Ficoll gradient as previously described (62).

### Antibodies and Reagents

Antibodies for detection of citrullinated histone H3 (R2 citrullination, Ab176843) and total histone H3 (Ab24834) were from Abcam (Cambridge, MA, USA). Anti-citrullinated fibrinogen antibody (20B2) was from ModiQuest (Oss, Netherlands). Antibody for detection of pan citrullination, F95 was from EMD Millipore (Billerica, MA, USA).

Recombinant human PAD2 and recombinant human PAD4 were generated in-house. Recombinant human histone H3 was from Cayman Chemicals (Ann Arbor, MI, USA).

DMEM without bicarbonate was from Agilent. DMEM with bicarbonate, RPMI, DPBS with 0.9mM calcium, HBSS, HEPES, glucose, 7.5% sodium bicarbonate, ionomycin, and 1M calcium chloride were from Invitrogen (Carlsbad, CA, USA).

### pH Level Adjustment of Media and Buffers

pH of all buffers and media were tested with pH meter (Thermo Fisher Scientific). For experiments with different pH levels, pH was adjusted with HCl and NaOH. In all other experiments, HEPES was used to adjust pH of buffers and media to 7.2. pH levels of buffers adjusted with HEPES at the start and the end of each experiment were tested (pH levels were between 7.0 and 7.4 after experiments).

### Western Blot Analysis

Equivalent amounts of cells were separated by SDS-polyacrylamide gel electrophoresis (4–12% gel) and then transferred to a nitrocellulose membrane. Membranes were then probed using anti-citrullinated histone H3 (Ab176843) and anti-total histone H3 (Ab24834) antibodies using the iBind (Invitrogen) system with 1:1,000 dilution. An HRP-conjugated anti-mouse IgG and anti-rabbit IgG were used as the secondary detection antibodies (1:5,000 dilution) before visualization of immunoreactive bands with an ECL reagent (Thermo Fisher). F95 western blot detection of pan citrullination used methods described before (57).

### Citrullination Assays

Recombinant human PAD2 (5 ng) or PAD4 (20 ng) was incubated with histone H3 (5 µg) in different media for 1 h at 37°C with 5% CO_2_. One million neutrophils were resuspended with 100 µl media and stimulated with 1 µM ionomycin for 30 min at 37°C. All reactions were performed in closed capped Eppendorf tubes and stopped with the addition of lithium dodecyl sulfate sample buffer (Invitrogen) and boiling.

### Fibrinogen Citrullination ELISA Assay

Nunc Maxisorp plates were coated with 1 µg/ml human fibrinogen overnight at 4°C. After blocking with PBS containing 1% BSA, rhuPAD2 and rhuPAD4 were titrated in Deimination buffer [40 mM Tris–HCl (pH 7.5), 5 mM NaCl, 1 mM DTT] and added to coated plates. After a 90-min incubation at 37°C with 5% CO_2_, anti-citrullinated Fibrinogen IgG (20B2) followed by rabbit anti-mouse IgG HRP (ab6728) were added to the plates followed by detection with a colorimetric substrate (TMB and Stop Solution, KPL). The absorbance was read on a plate reader at 450 nm and the data were analyzed using Softmax Pro software. Statistical analysis was performed with Prism GraphPad (GraphPad Software, La Jolla, CA, USA).

## Ethics Statement

Blood from healthy volunteers was obtained with informed consent under MedImmune, LLC’s blood donation program, and studies using human cells were performed in accordance with the Institutional Review Board guidelines.

## Author Contributions

YZ and GS generated ideas for this study. YZ and NM performed experiments and analyzed data. YZ, NM, and GS wrote the manuscript. All authors reviewed the manuscript for content, provided suggestions, and approved the final manuscript.

## Conflict of Interest Statement

YZ, NM, and GPS are full-time employees of MedImmune, a member of the AstraZeneca group. The reviewer ML and handling editor declared their shared affiliation.
